# Effects of Aerobic vs. Resistance Exercise on Vascular Function and Vascular Endothelial Growth Factor in Older Women

**DOI:** 10.3390/healthcare11182479

**Published:** 2023-09-07

**Authors:** Hyun-Bae Kim, Myong-Won Seo, Hyun Chul Jung

**Affiliations:** 1 Department of Korean Sports Medicine, Daegu Haany University, Gyeongsan-si 38610, Gyengsanbuk-do, Republic of Korea; hbkim@dhu.ac.kr; 2 Departments of Exercise Science, David B. Falk College of Sport and Human Dynamics, Syracuse University, Syracuse, NY 13244, USA; mseo04@syr.edu; 3 Sports Science Research Center, College of Physical Education, Kyung Hee University, Yongin-si 17104, Gyeonggi-do, Republic of Korea; 4 Department of Sports Coaching, College of Physical Education, Kyung Hee University, Yongin-si 17104, Gyeonggi-do, Republic of Korea

**Keywords:** aging, arterial stiffness, flow-mediated dilatation, regular exercise

## Abstract

This study aimed to investigate the effects of different types of exercise (aerobic vs. resistance) on vascular function and vascular endothelial growth factor in older women. Forty-three older women, aged 65–75 years old, voluntarily participated in this study. All participants were randomly assigned to one of the following three groups: aerobic exercise (AE; *n* = 14), resistance exercise (RE; *n* = 15), and control (CG; *n* = 14) groups. All participants in the exercise groups performed their respective exercises for 60 min/day, three days/week, for 16 weeks. The intensity of aerobic and resistance exercises was determined using the individual heart rate reserve (40–60%) and RPE (12–13), respectively. The vascular function test included the brachial-ankle pulse wave velocity (ba-PWV), carotid artery blood flow volume, and velocity. Participants’ blood samples were collected to analyze the vascular endothelial growth factor (VEGF). A significance level of 0.05 was set. Our results showed that ba-PWV improved following both AE (14.5%) and RE groups (11.1%) (all *p* < 0.05). Increases in carotid blood flow volume (AE: 15.4%, RE: 18.6%) and total artery peak velocity (AE: 20.4%, RE: 17%) were observed in AE and RE groups (*p* < 0.05), while flow total artery mean velocity (36.2%) and peak velocities (20.5%) were only increased in the aerobic exercise group (*p* < 0.05). VEGF was increased after resistance exercise (*p* < 0.05). Overall, aerobic exercise provides greater benefits on vascular function than resistance exercise but further research is needed on VEGF regarding whether this change is associated with vascular function improvement in older women.

## 1. Introduction

Aging is a time-associated deterioration in physical, physiological, and psychological functions under natural conditions [1]. The aging population has increased rapidly and become an important public health issue in developed countries such as South Korea [2]. According to Statistics Korea, the aging population aged 65 years old or more made up 17.5% in 2022 and will continue to increase to 20.6% in 2025 [3]. Age-related psychological dysfunctions include depressive and anxiety disorders [4] and vascular dysfunctions, which cause hypertension [5], endothelial dysfunction [6], coronary heart disease (CHD) [7], peripheral artery disease, and stroke [8]. It is a complex process to understand how aging affects psychological and vascular dysfunctions, but one possible mechanism involves the changes in central and peripheral blood flow [9].

Deficient blood flow in the brain, such as transient cerebral ischemia, has been reported in patients with generalized anxiety disorders [10], and increased pulse wave velocity (PWV), which results in vascular damage. Cerebral blood flow also declines with age, which may result in CVDs and hypertension in elderly individuals [11]. The PWV can be used as a prognostic indicator of vascular damage and increased PWV has been shown to contribute to the increased prevalence of hypertension and heart disease in elderly individuals [12,13]. Especially, the brachial-ankle PWV (ba-PWV) is a useful tool for assessing arterial stiffness and predicting cardiovascular disease risk. Therefore, blood flow, and ba-PWV, could be useful predictors of psychological and vascular dysfunction in elderly individuals.

Vascular endothelial growth factor (VEGF) was described as an angiogenic mitogen produced by endothelial cells [14]. VEGF is a vascular permeability factor that leads to changes in blood flow [15]. Physical exercise plays an important role in vascular function through its effects on VEGF [16,17]. Acute aerobic exercise has been shown to increase VEGF levels in elderly individuals with arterial pathologies [18]. In addition, Farzanegi et al. demonstrated that acute resistance training (i.e., upper- and lower-body) induces a favorable alteration in VEGF in physically inactive adults. Yet, there is no consensus on the optimal benefits of any physical exercise interventions (aerobic vs. resistance exercise) on VEGF concentration in elderly individuals, because of the variety of exercise conditions used in research studies. Furthermore, although Prior et al. suggested that the type, intensity, and duration of physical exercise are important for angiogenesis [19], there are no studies concerning optimal exercise programs for increasing angiogenesis through increasing VEGF levels [20]. Therefore, an examination of the effects of any physical exercise intervention type on vascular function and VEGF in the elderly is needed.

In women, the total number of CVDs was an estimated 275.2 million, and the age-standardized overall prevalence of CVDs was 6403 per 100,000 individuals per year [21]. Previous studies reported that women have a higher rate of cardiovascular mortality and a worse prognosis and morbidity process after an acute CV event compared with men [22,23,24]. Nevertheless, only a few studies have directly examined the effects of exercise type on psychological and vascular factors in older women. Therefore, this study investigates the effects of two different exercise types (aerobic vs. resistance) on vascular function and a vascular endothelial growth factor in older women. The present study hypothesized that exercise type (aerobic vs. resistant) would have different effects on vascular function and VEGF in older women.

## 2. Materials and Methods

### 2.1. Participants

Recruitment involved face-to-face interactions, distribution of flyers at community centers, and presentations to community organizations. Forty-three older women aged between 65 and 75 years were recruited from Suwon-si, Korea. The participants included individuals without any medications, orthopedic limitations, or cardiovascular, pulmonary, or metabolic diseases, as well as those without any signs of cardiovascular issues. A power analysis using G*Power program 3.1.9.2 (Dusseldorf, Germany) was used to determine the sample size required to detect within factors for a repeated-measures ANOVA. With an estimated statistical power of 0.80 and a significance level (alpha) of 0.05, a total sample size of 42 was determined to be necessary in order to detect an effect size of 0.25. The present study applied the random allocation sequence, enrolled participants, and allocated them to interventions. Participants were randomly assigned to one of three groups: aerobic exercise (AEG, *n* = 14), resistance exercise (REG, *n* = 15), or control (CG, *n* = 14) groups. All participants in the exercise groups performed their respective exercises for 60 min/day, three days/week, for 16 weeks. The participant was informed of the study procedure and the risk and benefits of this study, and a written consent form was obtained from the participant. This study was approved by the institutional review board of the Kyung Hee University (KHSIRB14-006). The present study was conducted following the ethical principles outlined in the 1964 Declaration of Helsinki, which constitutes the Code of Ethics established by the World Medical Association. The basic profiles of participants including age, height, and body mass are presented in Table 1. Measurement procedure was as follows: (1) anthropometric characteristics, (2) blood sample, and (3) *vascular function test.*

### 2.2. Vascular Endothelial Growth Factor

A blood sample was obtained two times at pre- (0 weeks) and post-test (17 weeks) to analyze the serum VEGF. Participants were asked to perform overnight fasting (at least 12 h) and were prohibited from severe physical activity at least 24 h before the test. Venous blood samples were collected from each participant between 8:00 and 9:00 am. The serum concentrations of human VEGF were determined using a commercial enzyme-linked immunosorbent assay kit (BIOO Scientific Corp., Austin, TX, USA), according to the manufacturer’s instructions.

### 2.3. Vascular Function Test

Vascular function test was conducted before and after 3-day intervention. Vascular stiffness was analyzed using brachial-ankle pulse wave velocity (ba-PWV), which was assessed using a noninvasive vascular screening device (VP-1000, Omron, Japan) following the manufacturer’s instructions. The participants rested for 10 min in the supine position before the examination. Plethysmographic sensors connected to the cuffs were wrapped around the brachia and ankles to simultaneously record the pulse volume waveforms. The time interval (DT) between the wavefronts of the brachial and ankle waveforms was determined. The distance from the brachium to the ankle (DL) was calculated using the height of the participant. The ba-PWV was calculated for each side using the following formula: ba-PWV (cm/s) = (¼ × [DL/DT]). The average of the left and right ba-PWV values was used for subsequent analyses. Transcranial Doppler ultrasonography (ClearVue 550, Philips, Amsterdam, The Netherland) was used to detect the blood volume and velocity of the common carotid artery (CCA). Transcranial Doppler ultrasonography was measured after vascular stiffness. After an initial 20 min rest in the supine position, the CCA was assessed using a 12.4 MHz linear transducer of the computed sonography system. Flow volume measurements, including flow total artery mean volume (FTAMV), peak systolic velocity (PSV), end-diastolic velocity (EDV), and total artery peak velocity (TAPV), were measured through B-mode ultrasonography 1.5–2 cm below the carotid bulb in the CCA.

### 2.4. Exercise Programs

The AEG followed an exercise program in which each exercise session was divided into a 10 min warm-up, 40 min exercise regimen, and 10 min cool-down. According to the guidelines of the American College of Sports Medicine [25], the intensity of aerobic exercise was determined using the heart rate reserve, which was set at 40–60% (moderate intensity). During the intervention period, heart rate was monitored monthly using portable heart rate monitors (RS400, Polar, Kempele, Finland) to ensure the maintenance of exercise intensity. The aerobic exercise program is presented in Table 2. The REG used the same basic protocol as that used for the AEG. Two different color Thera-Bands (yellow, 0.7–1.0 kg; red, 0.9–1.6 kg; Hygenic Corporation, Akron, OH, USA) and two sets of Dumbbells (1 and 1.5 kg) were used for resistance exercises. The resistance exercise consisted of two phases. The first phase, from the first to the eighth week, consisted of three sets of 10 repetitions per set; and the second phase, from the eighth to the sixteenth week, consisted of three sets of 15 repetitions per set. The intensity of resistance exercise was determined through rated perceived exertion (RPE; borg scale) [26], which was maintained at 12–13. All participants in the REG used the yellow band during the first phase and the red band during the second phase to maintain individual exercise intensity [27]. The resistance exercise program is presented in Table 2.

### 2.5. Statistical Analysis

Statistical analyses were performed using SPSS software (version 26.0). All data are presented as the mean ± standard deviation. A two-way analysis of variance with repeated measures was performed to determine the interaction effects of group and time on dependent variables, followed by a least-significant-difference post hoc analysis. A paired *t*-test was performed to analyze the main effect of time within the groups, whereas a one-way analysis of variance was performed to determine the main effect of the groups among the three groups. The Cohen’s *d* (effect size) was calculated for all variables between pre- and post-test. Statistical significance was set at *p* < 0.05.

## 3. Results

The changes in vascular stiffness are listed in Table 3. There was an interaction effect of group and time on the right-sided ba-PWV (F = 3.456, *p* < 0.05). The right-sided ba-PWV was decreased both in the AEG (*p* < 0.05) and REG (*p* = 0.05). However, no interaction effect was observed on the left-sided ba-PWV.

The changes in carotid artery blood flow volume and flow rates following different types of exercise intervention in older women are listed in Table 4. There were interaction effects of group and time for FV (F = 4.917, *p* < 0.05), FTAMV (F = 4.825, *p* < 0.05), TAPV (F = 4.062, *p* < 0.05), and PSV (F = 12.894, *p* < 0.001). In particular, FV in both the AEG (*p* < 0.01) and REG (*p* < 0.001) and FTAMV in the AEG (*p* < 0.01) were increased in the post-test compared to those in the pre-test. TAPV in both the AEG (*p* < 0.001) and REG (*p* < 0.005) was increased in the post-test compared to that in the pre-test. PSV in the CG (*p* < 0.001) was decreased, while it was increased in the AEG (*p* < 0.01) in the post-test compared to that in the pre-test. No interaction effect of time and EDV was observed in this study.

The change in VEGF following different types of exercise is presented in Table 5. There was an interaction effect of group and time for VEGF (F = 3.697, *p* = 0.035). In particular, VEGF in the REG (t = −2.275, *p* = 0.039) increased in the post-test compared to that in the pre-test.

## 4. Discussion

This study aimed to examine the effects of different types of exercise on vascular function and VEGF in older women. Our findings showed that participation in aerobic exercise was superior to provide a health benefit on vascular function compared to resistance exercise. Although VEGF did not change after the aerobic exercise intervention, resistance exercise using Thera-Bands and Dumbbells significantly increased VEGF.

Increased arterial stiffness is an age-related factor that increases the risk of various cardiovascular diseases [28] and therapeutic interventions such as exercise have been shown to modulate arterial stiffness [29]. The assessment of central and peripheral pulse wave velocity is the gold standard in vivo method for evaluating arterial stiffness [30]. In this study, both aerobic and resistance exercise for 16 weeks significantly decreased the right-sided ba-PWV. Our study demonstrated that both types of exercise have beneficial effects on vascular stiffness in older women. A previous study reported that short-term aerobic exercise has been shown to reduce arterial stiffness as observed by significant decreases in PWV in older adults, middle-aged participants with type 2 diabetes [31], and healthy participants [32], while long-term aerobic exercise has been shown to improve arterial stiffness in healthy older adults [33]. However, Kingsley et al. suggested that resistance exercise can lead to a temporary increase in arterial stiffness [34] and Kawano et al. reported a deterioration in arterial stiffness caused by resistance training in middle-aged individuals [35]. We speculate that resistance exercises using Thera-Bands and Dumbbells provide a safe and effective approach to target small muscle groups, potentially leading to a positive impact on arterial stiffness when compared to exercises involving large muscle groups. Further studies are required to compare the effects of different resistance exercise types (i.e., large muscle groups vs. small muscle groups) on arterial stiffness.

Blood volume and velocity are closely associated with arterial stiffness. Blood velocity is one of the most significant parameters used to identify carotid stenosis using Doppler ultrasonography [36]. In the present study, both aerobic and resistance exercise significantly improved FV and TAPV, whereas FTAMV and PSV improved in only aerobic exercise. The carotid artery, located in the neck, plays an important role in supplying oxygenated blood to the brain [37]. During physical exercise, the carotid artery dilates to allow for greater blood flow to the brain, ensuring that the brain receives an adequate supply of oxygen and nutrients [38,39]. Especially, aerobic exercise promotes the development of collateral circulation [40], which is a mechanism that helps maintain a steady blood flow to the brain, reducing the risk of a cardiac event [41]. Kingsley and Figueroa reported that resistance exercise for 12 weeks increased both resting and peak forearm blood flow in women (35–50 years-of-age) [42], and Egaña et al. showed that resistance exercise using an elastic band for 12 weeks enhanced calf blood flow in older women (59–79 years-of-age) [43]. In addition, Rossow et al. reported that high-intensity resistance exercise for 8 weeks increases the peak forearm blood flow in older participants, although this finding may not apply to all women, because of individual variations [12]. Our findings suggested that in older women, both aerobic and resistance exercises are effective in enhancing vascular function. However, not all variables of carotid artery blood flow volume and flow rate improved after 16 weeks of resistance exercise. A future long-term study (at least 24 weeks) is needed to confirm these findings.

In the present study, our findings showed that although VEGF significantly improved after 16 weeks of resistance exercise, aerobic exercise did not change VEGF in older women. The improvement in vascular structure and function is thought to be related to angiogenesis, which causes blood flow to increase. Angiogenesis is a complex process that occurs through the migration and proliferation of endothelial cells. Several studies have shown that VEGF plays an important role in angiogenesis [44], and regular physical exercise can promote angiogenesis by increasing VEGF levels. Recently, Vital et al. reviewed the association between physical exercise and VEGF in older individuals and suggested that the effect of physical exercise on serum VEGF levels showed conflicting results [17]. The findings from the present study align with previous research on the effects of resistance exercise. Shimizu et al. demonstrated that 4 weeks of resistance exercise improved VEGF levels in healthy older individuals [45]. Additionally, Alvar et al. reported that 5 weeks of resistance exercise enhanced angiogenesis, including increased VEGF expression, in adults [46]. Most studies used aerobic exercise, which resulted in significant increases in VEGF [18,47,48]; this could be due to the transfer of skeletal muscle VEGF into blood circulation [49]. Inconsistent with the aforementioned studies, our findings showed that aerobic exercise did not alter VEGF levels in older women. We speculate that the differing populations and the utilization of low exercise intensity in previous studies may have contributed to the disparate results regarding the effects of aerobic exercise. Therefore, further studies are needed to elucidate the association between aerobic exercise intensity and VEGF levels.

There are several limitations in this study. First, although the current ACSM guidelines recommend the RPE scale for older adults, this study applied the HRR-based aerobic exercise protocol that is equivalent to moderate-intensity exercise. The HRR-based exercise intervention is commonly used for most adults and some healthy older adults; thus, our study would meet the exercise recommendation (moderate intensity) for older adults. Second, exercise intensity was monitored every 4 weeks, which may affect the daily-based workout rate. Third, the diets were not controlled throughout the intervention period. Dietary choices may affect serum VEGF levels in older individuals. In a future study, the exercise program needs to be designed more systematically to provide more healthy benefits among older adults.

## 5. Conclusions

Our study confirmed that aerobic exercise provides greater benefits on vascular function than resistance exercise but further research is needed on VEGF regarding whether this change is associated with vascular function improvement in older women.

## Figures and Tables

**Table 1:** Anthropometric characteristics of participants.

Variables	Aerobic (*n* = 14)	Resistance (*n* = 15)	Control (*n* = 14)	*p*-Value
Age (yr)	68.4 ± 3.51	68.3 ± 3.45	67.5 ± 5.17	0.64
Height (cm)	152.6 ± 4.45	152.1 ± 3.88	151.0 ± 3.14	0.19
Body mass (kg)	56.4 ± 5.35	56.1 ± 4.03	57.1 ± 6.72	0.79

**Table 2:** Aerobic and resistance exercise program for older women.

Type		Exercise Program	Intensity	Duration
	Warm-up	Stretching	Heart rate reserve 40–60%	10 min
Aerobic exercise	Main exercise	Walking, Knee-up, Low front kick, Low back kick, One step, Two-step, Side touch, March, Turn, Change, Grapevine step, Step-touch (one-step, side-step), Open step, Lunge, Mambo, Chachacha, Shasse (two-step, triple-step)		40 min
	Cool-down	Stretching		10 min
Resistance	Warm-up	Stretching		10 min
	Main exercise	Thera-Band Ankle: plantar-flexion, ankle- dorsi-flexion, eversion and inversion Knee: leg press, knee extension, knee flexion Hip: hip extension, hip flexion, hip abduction, hip adduction Abdomen: hip extension, hip flexion, hip abduction, hip adduction Back: Hyper-extension Arm: elbow flexion, elbow extension	RPE 12–13	40 min
		Dumbbell Dumbbell lateral raise, Bent-over dumbbell raise, Dumbbell internal rotation, Dumbbell shrug, Dumbbell curl, Dumbbell, Kickback, Hammer curl, Lunge, Dumbbell side bend, Dumbbell stiff-leg dead lift		
	Cool-down	Stretching		10 min

**Table 3:** Changes in vascular stiffness following different type of exercise intervention in older women.

Variables	Group	Pre-Test	Post-Test	ES		*p*-Value
Right ba-PWV (cm·s^−1^)	AEG	1669.92 ± 328.64	1427.00 ± 200.71 *	0.92	G	0.039
	REG	1542.27 ± 272.99	1371.27 ± 119.65 *	0.87	T	0.008
	CG	1656.36 ± 287.76	1695.14 ± 329.75	0.12	G × T	0.042
Left ba-PWV (cm·s^−1^)	AEG	1695.00 ± 402.03	1525.23 ± 247.48	0.52	G	0.121
	REG	1536.87 ± 272.54	1421.20 ± 243.38	0.45	T	0.240
	CG	1632.71 ± 283.02	1732.43 ± 394.38	0.29	G × T	0.096

Note: All values are mean (M) ± standard deviation (SD). ba-PWV, brachial-ankle pulse wave velocity; AEG, aerobic exercise group; REG, resistance exercise group; CG, control group; ES, Cohen’s *d*, * indicates significant difference between pre-test and post-test.

**Table 4:** Changes in carotid artery blood flow volume and flow rate following different types of exercise intervention in older women.

Variables	Group	Pre-Test	Post-Test	ES		*p*-Value
FV (L·min^−1^)	AEG	0.3316 ± 0.07	0.3820 ± 0.07 *	0.72	G	0.842
	REG	0.3244 ± 0.07	0.3846 ± 0.08 *	0.80	T	0.001
	CG	0.3452 ± 0.11	0.3382 ± 0.10	0.07	G × T	0.013
FTAMV (cm·s^−1^)	AEG	16.66 ± 6.04	22.61 ± 3.37 *	1.26	G	0.084
	REG	16.90 ± 4.13	18.45 ± 2.74	0.45	T	0.001
	CG	16.12 ± 3.82	16.72 ± 5.23	0.13	G × T	0.014
TAPV (cm·s^−1^)	AEG	27.01 ± 4.97	32.54 ± 4.91 *	1.11	G	0.223
	REG	30.00 ± 5.30	35.01 ± 4.37 *	1.04	T	<0.001
	CG	29.15 ± 7.41	29.40 ± 7.22	0.03	G × T	0.025
PSV (cm·s^−1^)	AEG	61.08 ± 16.79	75.26 ± 8.17 *	1.14	G	0.022
	REG	63.09 ± 16.72	71.39 ± 13.15	0.06	T	0.302
	CG	65.24 ± 12.27	50.49 ± 7.41 *	1.50	G × T	<0.001
EDV (cm·s^−1^)	AEG	18.57 ± 5.56	20.02 ± 3.77	0.31	G	0.854
	REG	17.61 ± 3.15	20.44 ± 3.23	0.89	T	0.006
	CG	17.65 ± 4.63	19.37 ± 4.90	0.36	G × T	0.681

Note: AEG, aerobic exercise group; REG, resistance exercise group; CG, control group; FV, flow volume; FTAMV, flow total artery mean velocity; TAPV, total artery peak velocity; PSV, peak systolic velocity; EDV, end-diastolic velocity; ES, Cohen’s *d*. * Indicates significant difference between pre-test and post-test.

**Table 5:** Change in VEGF following different types of exercise intervention in older women.

Variable	Group	Pre-Test	Post-Test	ES	*p*-Value	
VEGF	AEG	56.72 ± 28.91	76.39 ± 17.39	0.84	G	0.026
	REG	40.38 ± 21.16	58.51 ± 23.37 *	0.81	T	0.291
	CG	76.01 ± 28.46	58.40 ± 37.41	0.53	G × T	0.035

Note: VEGF, vascular endothelial growth factor; AEG, aerobic exercise group; REG, resistance exercise group; CG, control group; ES, Cohen’s *d*. * Indicates significant difference between pre-test and post-test.

## Data Availability

Data used in this study are available upon request.
